# The Association Between Mobile App Use and Change in Functional Capacity Among Cardiac Rehabilitation Participants: Cohort Study

**DOI:** 10.2196/44433

**Published:** 2023-05-15

**Authors:** Janah May Oclaman, Michelle L Murray, Donald J Grandis, Alexis L Beatty

**Affiliations:** 1 School of Medicine University of California, San Francisco San Francisco, CA United States; 2 Division of Cardiology Department of Medicine University of California, San Francisco San Francisco, CA United States; 3 Department of Epidemiology and Biostatistics University of California, San Francisco San Francisco, CA United States

**Keywords:** cardiac rehabilitation, mobile application, functional capacity, blood pressure, telemedicine, mHealth, telehealth assessment, e-health, youth, adolescence, EHR, electronic health record

## Abstract

**Background:**

Cardiac rehabilitation (CR) is underused in the United States and globally, with participation disparities across gender, socioeconomic status, race, and ethnicities. The pandemic led to greater adoption of telehealth CR and mobile app use.

**Objective:**

Our primary objective was to estimate the association between CR mobile app use and change in functional capacity from enrollment to completion in patients participating in a CR program that offered in-person, hybrid, and telehealth CR. Our secondary objectives were to study the association between mobile app use and changes in blood pressure (BP) or program completion.

**Methods:**

We conducted a retrospective cohort study of participants enrolled in CR at an urban CR program in the United States. Participants were English speaking, at least 18 years of age, participated in the program between May 22, 2020, and May 21, 2022, and downloaded the CR mobile app. Mobile app use was quantified by number of exercise logs, vitals logs, and education material views. The primary outcome was change in functional capacity, measured by change in 6-minute walk distance (6MWD) from enrollment to completion. The secondary outcome was change in BP from enrollment to completion. We estimated associations using multivariable linear or logistic regression models adjusted for age, sex, race, ethnicity, socioeconomic status by ZIP code, insurance, and primary diagnosis for CR referral.

**Results:**

A total of 107 participants (mean age 62.9, SD 13.02 years; 90/107, 84.1% male; and 57/105, 53.3% self-declared as White Caucasian) used the mobile app and completed the CR program. Participants had a mean 64.0 (SD 54.1) meter increase in 6MWD between enrollment and completion (*P*<.001). From enrollment to completion, participants with an elevated BP at baseline (≥130/80 mmHg) experienced a significant decrease in BP (systolic BP –11.5 mmHg; *P*=.002 and diastolic BP –7.7 mmHg; *P*=.003). We found no significant association between total app interactions and change in 6MWD (coefficient –0.03, 95% CI –0.1 to 0.07; *P*=.59) or change in BP (systolic coefficient 0.002, 95% CI –0.03 to 0.03; *P*=.87 and diastolic coefficient –0.005, 95% CI –0.03 to 0.02; *P*=.65). There was no significant association between total exercise logs and change in 6MWD (coefficient 0.1, 95% CI –0.3 to 0.4; *P*=.57) or total BP logs and change in BP (systolic coefficient –0.02, 95% CI –0.1 to 0.06; *P*=.63 and diastolic coefficient –0.02, 95% CI –0.09 to 0.04; *P*=.50). There was no significant association between total app interactions and completion of CR (adjusted odds ratio 1.00, 95% CI 0.99-1.01; *P*=.44).

**Conclusions:**

CR mobile app use as part of an in-person, hybrid, or telehealth CR program was not associated with greater improvement in functional capacity or BP or with program completion.

## Introduction

Cardiovascular disease remains the leading cause of death globally and in the United States for men, women, and most racial and ethnic groups [1,2]. The United States spent US $320.1 billion in cardiovascular-related costs in 2016, with an overall increase in spending driven mostly by inpatient and ambulatory care [3,4]. Cardiac rehabilitation (CR) is a multidisciplinary exercise training customized to help patients with heart disease to recover, improve, and prevent future cardiac events [5-7]. Exercise-based CR studies have shown reductions in mortality and hospital admissions as well as improvement in quality of life [5-7]. However, CR is highly underused around the globe [7-9]. Globally, uptake of eligible patients ranges from 10% to 60%, with an uptake of 24% in the United States [7,10-14]. With barriers such as transportation, cost, and participation disparities across sex, gender, socioeconomic status (SES), race, and ethnicity, new delivery strategies are needed to increase accessibility and participation in CR [7,8,13,15].

The role of digital devices has significantly increased with the increasing trend of digitalization [16-18]. As mobile phone use increases, advancement in mobile technology and its application have the potential to transform health care delivery and support vulnerable populations [18-20]. Mobile health apps can organize and present relevant information and facilitate clinician-patient communication [16,21]. Prior studies have shown the potential of mobile health apps in transforming CR, showing that it is feasible to use mobile health apps to help deliver CR. [22-24]. With the COVID-19 pandemic, programs began to implement telehealth and hybrid approaches to delivering care, including the use of mobile apps [25,26]. However, it is unclear whether the extent of engagement with mobile apps is associated with important CR outcomes, such as functional capacity or blood pressure (BP).

The primary objective of this study was to estimate the association between CR mobile app use—measured by total app interactions—and change in functional capacity—measured by change in 6-minute walk distance (6MWD)—between enrollment and completion. We hypothesized that among patients using a CR mobile app, greater mobile app use would be associated with greater change in 6MWD. Secondary objectives included studying the association between total app interactions and changes in BP, physical activity logs and change in 6MWD, frequency of BP logs and change in BP, as well as the association between total app interactions and completion of CR. The study has the potential to quantify the impact of a mobile app on clinical outcomes in CR programs.

## Methods

### Design and Participants

We conducted a retrospective cohort study of adult participants enrolled in CR at University of California, San Francisco (UCSF) CR from May 22, 2020 (when use of the mobile app began) until May 21, 2022. For the primary study analysis, we included all participants aged ≥18 years who were enrolled in CR and downloaded the mobile app. Since the mobile app was only available in English, the study only included English-speaking participants. For the primary outcome analysis, we included only participants with 6MWD measurements at both enrollment and completion. For participants who enrolled in CR multiple times during this period (n=16), we analyzed the first episode of participation in CR.

### CR Program and Mobile App

**Figure 1:**
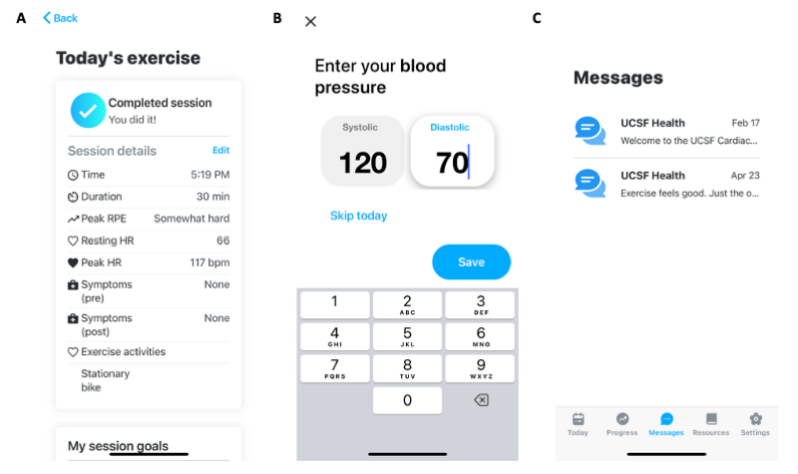
Screenshots of a mobile app used by patients in a retrospective cohort study of cardiac rehabilitation patients at University of California, San Francisco from May 22, 2020, to May 21, 2022. Features of the mobile app include (A) exercise log, (B) blood pressure log, and (C) chat with staff.

Participants enrolled in CR at UCSF can participate in in-person, telehealth, or hybrid CR [26]. Use of a mobile app (Chanl Health; Figure 1) is offered to all CR patients in all modes of participation but is optional. The mobile app allows patients to log exercise activities and vitals, view educational materials, set medication reminders, and message CR staff. CR staff include doctors, nurses, exercise physiologists, pharmacists, a nutritionist, and a mental health provider. Concomitantly, CR staff can view patient logs and activities. Eligible patients were informed of the mobile app and taught how to use the app by a CR staff member using task-based training on entering vital signs, entering an exercise session, viewing an education module, messaging with providers, and setting up medication reminders (if desired). There was no cost to the patient associated with using the app.

### Mobile App Use

Patient app interaction was retrieved from mobile app use logs. Patient app interactions included exercise logs, vital sign logs (eg, BP, weight, blood glucose, and oxygen saturation), education material views, and chat with providers. Data were manually entered into the app by patients. Total app interaction was measured as the sum of total exercise logs, vital sign logs, and education material views. For analysis of BP measurements recorded in the app, when multiple measurements were present with the same date and time, B*P* values were averaged.

### Outcomes

The primary outcome was change in functional capacity between enrollment and completion of the CR program, measured by change in 6MWD. At enrollment and completion, participants completed a 6-minute walk test on a standard course at the UCSF CR center [27]. The secondary outcome was change in BP between enrollment and completion of the CR program. At enrollment and completion, systolic and diastolic BP were measured with a standard BP cuff at the UCSF CR center. Measurements of 6MWD and BP were recorded by CR staff in CR Quality Improvement data and entered into the electronic health records, which served as the data sources for outcome measurements.

### Other Measurements

Participant characteristics were obtained from structured electronic health record data, including age, sex assigned at birth, race, ethnicity, insurance, SES by zip code, and primary reason for CR referral. Sex assigned at birth was used since it was the only available data. Insurance was categorized as either private or public (eg, Medicare, Medicaid, and other city- or state-sponsored health plans). SES by zip code was established using the UCSF Health Atlas, where zip codes across California are rated between 1 (lowest quintile) to 5 (highest quintile) of socioeconomic advantage [28]. The UCSF Health Atlas uses neighborhood data system from a census tract-level data accounting for 7 factors (eg, percentage of people aged ≥25 years with college, high school, or less than high school education level; percentage of people with blue-collar jobs; percentage of people aged ≥16 years in the workforce, but without a job; and percentage of people below 200% of the federal poverty level, median household income, and median rent) [28]. The primary diagnosis associated with the CR referral was used as the primary reason for referral, categorized as coronary artery disease, valvular disease, heart failure, or other diseases. If a coded referral diagnosis was not present, reason from referral was determined from chart review of the patient’s individualized treatment plan for CR.

Clinical CR measurements at enrollment and completion were obtained from CR Quality Improvement data, including BMI, waist-to-hip ratio, General Anxiety Disorder (GAD)-7, and Patient Health Questionnaire (PHQ)-9. BMI was calculated through weight and height recorded in the health electronic record, and waist-to-hip ratio were measured in person by CR staff using standardized methods. PHQ-9 was scored 0-27, with higher scores representing more severe depressive symptoms; GAD-7 was scored 0-21, with higher scores representing more severe anxiety symptoms. Laboratory test values were obtained from structured electronic health record data, including hemoglobin A_1c_, total cholesterol, low-density lipoprotein, high-density lipoprotein, and triglycerides. Enrollment laboratory values were defined as the measurements prior to the participant’s first day in the CR program. Completion laboratory values were defined as a recorded measurement 3 to 6 months after enrollment.

### Statistical Analysis

We included all patients within the study period who fit our inclusion criteria; therefore, sample size was not calculated a priori. Patient characteristics, app interactions, and clinical test results were summarized using descriptive statistics, mean (SD) for normally distributed characteristics, and median (IQR) for nonnormally distributed characteristics. Changes in clinical characteristics were calculated by subtracting enrollment values from completion values and compared using a paired *t* test (2-tailed). Linear regression was used to evaluate the association between change in 6MWD and total app interactions, change in 6MWD and total exercise logs, change in BP and total app interactions, and change in BP and total BP logs. We also examined, among all people enrolled in CR who downloaded the app, whether total app interactions were associated with completion of CR using logistic regression. Regression models were adjusted for confounders including age, sex assigned at birth, race, ethnicity, SES by zip code, insurance, and primary diagnosis. Significance was considered *P*<.05. With our sample size, we would have >70% power to detect a difference of 25 meters (the minimum clinically important difference) per 100 app uses [29]. All statistical analyses were performed using Stata (version 17.0; StataCorp).

### Ethics Approval

This study was approved by the UCSF Institutional Review Board (21-33754), who waived the requirement of informed consent for this retrospective study. All identifiable data were stored on secure cloud servers. From identifiable raw data, deidentified data sets were created for analysis. There was no compensation to patients.

## Results

Between May 22, 2020, and May 21, 2022, a total of 107 adult English-speaking patients enrolled and completed the UCSF CR program and downloaded the CR mobile app. Mean participant age was 62.9 (SD 13.02) years, with 90/107 (84.1%) participants having male sex assigned at birth, 57/105 (53.3%) self-declared as White Caucasian, 96/105 (91.4%) self-declared as non-Hispanic, 75/107 (70.1%) with private insurance, and 62/106 (58.5%) categorized as the highest quintile of SES by zip code (Table 1) . The leading primary reason for CR referral was coronary artery disease with 76/107 (71.0%) participants.

Participants had a mean 64.0 (SD 54.1; *P*<.001) meter increase in 6MWD between enrollment and completion (Table 2). Among all participants, there was not a significant change in systolic BP or diastolic BP from enrollment to completion. However, participants with an elevated BP at baseline (³130/80 mmHg) experienced a significant decrease in BP from enrollment to completion (Table 2).

**Figure 2:**
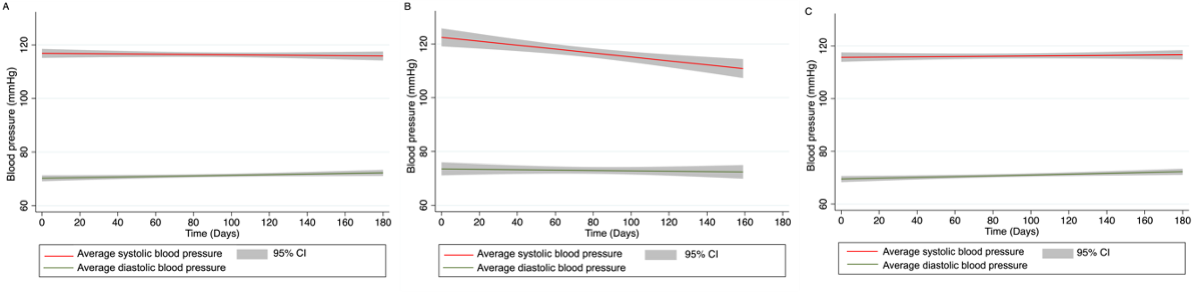
Average blood pressure over time of patients using a mobile app in a retrospective cohort study of patients in cardiac rehabilitation at University of California, San Francisco from May 22, 2020, to May 21, 2022. (A) Average blood pressure of all patients at enrollment (n=101). (B) Average blood pressure of patients with elevated blood pressure (≥130/80 mmHg) at enrollment (n=26). (C) Average blood pressure of patients with normal blood pressure (<130/80 mmHg) at enrollment (n=75).

Participants had a median of 92 (IQR 20-200) total app interactions, a median of 19 (IQR 1-57) exercise logs, and a median of 26 (IQR 4-67) BP logs. Of 107 participants, 17 (15.9%) had no exercise logs, 4 (3.7%) had no app interactions (only downloaded the app), and 8 (7.5%) had no BP logs. Logged BPs were stable over time among all participants but demonstrated a decrease over time among participants with initially elevated BP (n=26; Figure 2).

Evaluating the association between total app interactions and change in 6MWD with a linear regression model adjusted for age, sex, race, ethnicity, SES by zip code, insurance, and primary diagnosis revealed no significant association (Table 3). There was no association between total exercise logs and change in 6MWD. Similarly, we did not observe associations between total app interactions or total BP logs and change in BP (Table 3).

We conducted a sensitivity analysis examining the adjusted association between chat messages and change in functional capacity, finding no association (coefficient 0.48, 95% CI –0.84 to 1.80; *P*=.47). Adding chat messages to total app interactions did not meaningfully change the adjusted association between total app interactions and functional capacity (coefficient –0.04, 95% CI –0.1 to 0.06; *P*=.43). Representing total app interactions as quartiles of use also demonstrated no association between quartile of app interactions and functional capacity.

Analyzing the data of 149 patients, who were ≥18 years of age, enrolled in the CR program between May 22, 2020, and February 21, 2022 (since program completion of patients has an average of 4 months), and downloaded the mobile app, we found no significant association between total app interactions and program completion (odds ratio 1.00; 95% CI 0.99-1.00; *P*=.10), even after adjustment for age, sex, race, ethnicity, SES by zip code, insurance, and primary diagnosis (odds ratio 1.00; 95% CI 0.99-1.01; *P*=.44).

**Table 1:** Characteristics of patients using a mobile app in a retrospective cohort study of patients in cardiac rehabilitation at University of California, San Francisco from May 22, 2020, to May 21, 2022.

Characteristics		Values
Age in years (n=107), mean (SD)		62.9 (13.02)
**Sex at birth (n=107), n (%)**		
	Male	90 (84.1)
	Female	17 (15.9)
**Race (n=105), n (%)**		
	African American	2 (1.9)
	Asian	29 (27.6)
	Pacific Islander	1 (1)
	White Caucasian	57 (53.3)
	Multirace	4 (3.7)
	Others	12 (11.4)
**Ethnicity (n=105), n (%)**		
	Hispanic	9 (8.6)
	Non-Hispanic	96 (91.4)
**Socioeconomic status** **by** **zip code (n=106), n (%)**		
	1 (lowest)	0 (0)
	2	2 (1.9)
	3	11 (10.4)
	4	31 (29.3)
	5 (highest)	62 (58.5)
**Insurance (n=107), n (%)**		
	Public insurance	32 (29.9)
	Private insurance	75 (70.1)
**Primary diagnosis (n=107), n (%)**		
	Coronary artery disease	76 (71)
	Heart failure	13 (12.2)
	Valvular	14 (13.1)
	Other	4 (3.7)
**Mobile app use (n=107), median (IQR)**		
	Total app interactions	92 (20-200)
	Total exercise logs	19 (1-57)
	Total blood pressure logs	26 (4-67)

**Table 2:** Clinical results of patients using a mobile app in a retrospective cohort study of patients in cardiac rehabilitation at University of California, San Francisco from May 22, 2020, to May 21, 2022 (n_e_ represents number of participants at enrollment, and n_c_ represents number of participants at completion).

Characteristics	Enrollment	Completion	Change	*P* value
6-minute walk distance (meters; n=107), mean (SD)	447.1 (85.1)	510.8 (99.3)	64 (54.1)	<.001
Elevated BP^a^ (³130/80 mmHg; n_e_=101; n_c_=67), n (%)	26 (25.7)	14 (20.9)	N/A^b^	N/A
Systolic BP (mmHg; all patients; n_e_=101; n_c_=67), n (%)	119.5 (11.7)	117.2 (12)	–2.7 (11.9)	.07
Diastolic BP (mmHg; all patients; n_e_=101; n_c_=67), n (%)	73.9 (7.6)	71.6 (9.4)	–2 (8.3)	.06
Systolic BP (<130/80 mmHg at enrollment; n_e_=75; n_c_=50), n (%)	115.1 (8.3)	116.4 (12.6)	0.2 (10.2)	.92
Diastolic BP (<130/80 mmHg at enrollment; n_e_=75; n_c_=50), n (%)	70.9 (5.8)	70.8 (9.3)	–0.1 (7.1)	.90
Systolic BP (≥130/80 mmHg at enrollment; n_e_=26; n_c_=17), n (%)	132.4 (10.6)	121.1 (10.7)	–11.5 (12.4)	.002
Diastolic BP (≥130/80 mmHg at enrollment; n_e_=26; n_c_=17), n (%)	82.5 (80.1 to 86.7)	74.8 (65.5 to 81.1)	–7.7 (–14 to –0.4)	.003
BMI (kg/m^2^; n=107), n (%)	26.7 (4.4)	26.9 (4.5)	0.3 (1.4)	.03
Waist-to-hip ratio (n=107), n (%)	0.9 (0.07)	0.9 (0.08)	0 (0)	<.001
GAD-7^c^ (n=107), median (IQR)	1 (0 to 5)	1 (0 to 3)	–1.2 (–3 to 0)	<.001
PHQ-9^d^ (n=107), median (IQR)	3 (1 to 6)	1 (0 to 3)	–1.9 (–3 to 0)	<.001
Hemoglobin A_1c_ (%; n_e_=60; n_c_=33), median (IQR)	5.6 (5.3 to 5.9)	5.6 (5.4 to 6.1)	–0.1 (–0.2 to 0.3)	.70
Total Cholesterol (mg/dL; n_e_=68; n_c_=46), n (%)	154.7 (47.8)	134.2 (45.1)	–22.5 (51.3)	.01
LDL^e^ (mg/dL; n_e_=67; n_c_=44), n (%)	89.2 (41)	74.4 (35.7)	–21.7 (43.8)	.006
HDL^f^ (mg/dL; n_e_=68; n_c_=45), n (%)	43.7 (13.7)	45.4 (16.4)	3.9 (16.6)	.16
Total triglyceride (mg/dL; n_e_=72; n_c_=45), n (%)	107.9 (52.2)	94.4 (42.9)	–10.2 (53.3)	.25

^a^BP: blood pressure.

^b^N/A: not applicable.

^c^GAD-7: General Anxiety Disorder-7.

^d^PHQ-9: Patient Health Questionnaire-9.

^e^LDL: low-density lipoprotein.

^f^HDL: high-density lipoprotein.

**Table 3:** Associations between clinical outcomes and mobile app use in patients using a mobile app in a retrospective cohort study of patients in cardiac rehabilitation at University of California, San Francisco from May 22, 2020, to May 21, 2022 (models were adjusted for age, sex assigned at birth, race, ethnicity, socioeconomic status by zip code, insurance, and primary diagnosis).

Clinical outcome			Coefficient (95% CI)		*P* value
**Change in 6-minute walk distance**					
	Total app interactions	–0.03 (–0.1 to 0.07)		.59	
	Total exercise logs	0.1 (–0.3 to 0.4)		.57	
**Change in systolic blood pressure**					
	Total app interactions	0.002 (–0.03 to 0.03)		.87	
	Total blood pressure logs	–0.02 (–0.1 to 0.06)		.63	
**Change in diastolic blood pressure**					
	Total app interactions	–0.005 (–0.03 to 0.02)		.65	
	Total blood pressure logs	–0.02 (–0.09 to 0.04)		.50	

## Discussion

### Principal Results

In this study of 107 patients participating in CR with the use of a mobile app, we found no association between the amount of app use and improvement in clinical outcomes, including 6MWD and BP. We also did not observe an association between app use and completion of CR.

### Comparison With Prior Work

Previous research has shown that CR programs using mobile apps can achieve similar or improved outcomes compared to traditional CR programs [26]. However, there has been a lack of published research on associations between patient interaction with CR mobile apps and clinical outcomes. In their study, Widmer et al [30] found that digital health interventions in which patients interact with the app through reporting dietary and exercise habits showed a nonsignificant reduction in cardiovascular-related rehospitalizations [30]. In a randomized study, Rosario et al [31] found no statistically significant difference in 6MWD, waist measurement, RPE, and resting heart rate comparing hospital-based CR patients with and without a smart phone app, portable BP monitor, and weight scale [31]. In our nonrandomized study, we found no association between total app interactions and change in functional capacity (change in 6MWD) or change in BP.

In a systematic review of 6 randomized control trials of CR mobile apps, Tuttle et al [32] found inconsistent clinical results. Analysis suggested improved outcomes with apps that incorporated automated data collection, such as automatic step counters, automatic information logs, correctional goal setting, and real-time feedback [32]. Meanwhile, apps without automatic data collection and feedback were not successful [32]. The app used by patients in this study required manual data entry and did not provide automated feedback; moreover, its use was not associated with improved outcomes, which is consistent with the systematic review findings.

Nonetheless, with the larger use of smartphones comes an inherent shift of mobile app incorporation into clinical care. In addition, there is a high interest of mobile app use for health care delivery among patients and clinicians [9,33]. With this interest, technologies such as mobile apps can provide flexibility, scalability of care, accessibility of information, and communication among teams and patients [16,20-23]. As we have learned during the COVID-19 pandemic, mobile apps provided a mechanism to facilitate CR care and information exchange between patients and providers in a hybrid and remote setting, with no significant difference in clinical outcomes from in-person CR programs [26]. Patient experience is considered an important predictor for health care quality affecting clinical outcomes, medical adherence, and hospital retention [34-37]. Similarly, provider experience, although less studied, has been shown to affect patient outcomes and successful integration of programs [36,38]. However, little research is done on the role of mobile apps in improving patient and provider experiences [39,40]. This study did not capture measures of patient and provider satisfaction with mobile app use. Future studies should examine the impact of mobile app use on patient and provider experience.

### Limitations

Certain limitations must be considered. Generalizability may be limited since the cohort was enrolled and completed the program during the COVID-19 pandemic. Other factors that may affect generalizability include underrepresentation across sex assigned at birth, race, ethnicity, and SES since the cohort was mostly male, White Caucasian, non-Hispanic, living in neighborhoods associated with high SES, and with private insurance. SES by zip code incompletely captures individual-level SES factors.

This study only examined mobile app use among people who chose to download a mobile app and cannot account for differences between this population and people who did not choose to download the mobile app. Moreover, the patient population was only from a single center with a limited sample size. Additionally, in this observational study, using data collected during the routine course of health care, we could not account for all potential unmeasured confounders.

### Conclusions

In a population of patients participating in CR and using a mobile app, this study found no associations between total app interactions and changes in functional capacity, total exercise logs and changes in functional capacity, total app interactions and program completion, total app interactions and changes in BP, and total BP logs and changes in BP. Mobile app use was not associated with completion of the program. Given the lack of association between mobile app use and clinical outcomes, we encourage further study of the impact of mobile app use on patient and provider experience. Importantly, this research should be conducted in a diverse population to address existing disparities.

## Abbreviations

6WMD: 6-minute walk distance
BP: blood pressure
CR: cardiac rehabilitation
GAD-7: General Anxiety Disorder
PHQ-9: Patient Health Questionnaire (PHQ)-9
SES: socioeconomic status
UCSF: University of California, San Francisco

## Data Availability

Since data derive from identifiable data and there is a risk of reidentification, access to data sets is limited. A deidentified data set may be available upon request and execution of a data use agreement.
